# Vitreous Substitutes: From Tamponade Effect to Intraocular Inflammation

**DOI:** 10.1155/2014/159832

**Published:** 2014-09-01

**Authors:** Mario R. Romano, Xun Xu, Kenneth K. W. Li

**Affiliations:** ^1^Department of Neuroscience, University Federico II, Via Pansini 5, 80131 Naples, Italy; ^2^Department of Ophthalmology, Shanghai First People's Hospital Affiliated to Shanghai Jiaotong University, Shanghai, China; ^3^Department of Ophthalmology, United Christian Hospital and Tseung Kwan O Hospital, Hong Kong; ^4^Department of Ophthalmology, LKS Faculty of Medicine, The University of Hong Kong, Hong Kong

Vitreous substitutes have been developed both as an intraoperative and as a postoperative tool for the surgical treatment of complicated vitreoretinal diseases. The tamponade effect of the vitreous substitutes depends on the arc of contact between the agent and the inner retinal surface, which mainly depends on 4 physical parameters, namely, specific gravity, buoyancy, interfacial tension, and viscosity. As reported by F. Barca et al., the choice of different intraocular tamponade agents depends on the location of retinal break(s), compliance of postoperative posture, type of vitreoretinal disease(s), and duration required for the tamponade. Due to the hydrophobic property of intraocular tamponade agent, a thin aqueous layer invariably exists between the tamponade agent and the retina. This thin aqueous layer is further exaggerated in highly myopic eyes with posterior staphyloma to become a pocket of fluid leading to a theoretical reduction of the tamponade effect. However, the debate remains open on whether it is still worthwhile to use silicone oil in these eyes as X. Valldeperas and J. Lorenzo-Carrero reported contradicting results. An increased improved anatomical success using silicone oil in highly myopic eyes in their study was probably due to the lower shear retinal stress of the compartmentalized fluid, scarcely influenced by ocular movements, allowing the macular hole to close and the retinal detachment to reattach.

Based on this hypothesis, K. Isakova et al. reported a theoretical model that predicted the stability conditions of the interface between the aqueous and a vitreous substitute. They showed that the presence of a thin layer of aqueous between the retina and intraocular tamponade is responsible for significant reduction in the retinal shear stress. Their model also explains the instability of the interface leading to the formation of intraocular emulsion that remains the main drawback of the use of intraocular tamponade agents. Although the tolerance for vitreous substitutes remains generally good, the recent introduction of new mixed compounds, such as heavy silicone oil (HSO), has been associated with relatively high complication rates. In particular, emulsification and severe inflammatory reactions can lead to poor functional prognosis. According to L. Ambrosone et al., the spontaneous formation of water-silicone oil is a rare event and the very low concentration of surface-active agents cannot account for the systematic production of emulsions. The authors suggested that gravitational instability, originated at the interface by tangential disturbances, plays a more significant role in the formation of emulsions.

Semifluorinated compounds and perfluorocarbon liquids (PFCLs), mainly used only as intraoperative tamponades, are more prone to induce inflammation and emulsification.

As reported by M. S. Figueroa and D. R. Casas, PFCLs have also been used as postoperative short-term tamponade agent with development of up to 30% inflammation and retinal infiltration due to foreign-body reaction, sustained by macrophages that phagocytosed the PFCL droplets. Q. Yu et al. suggested that the physical properties of these tamponade agents, mainly the low viscosity and surface tension, reduce their stability and the superficial forces and could lead to significant clinical findings such as intraocular inflammation, raised intraocular pressure (IOP), and sticky oil formation.

From a retrospective study conducted on 100 eyes, H. Schwarzer et al. concluded that HSO does not induce alarming complications in the majority of cases. However, they suggested that long-term tamponade with HSO will require more frequent follow-up because of its high incidence of IOP elevations or intraocular inflammation. In fact D. Odrobina and I. Laudańska-Olszewska reported, at 3 months after the surgery, the topographic evidences of persistence of small hyperreflective round shaped SO droplets above the optic nerve and in the cystoid retinal spaces. Despite good anatomical success rate with HSO, J. Prazeres et al. reported a 52% rate of emulsification and 40% rate of keratic precipitates in their series with a 16% incident of IOP elevation. Interestingly F. Morescalchi et al. also reported more disturbing complications of HeavySil, a combination of high purity 75% silicone oil 5000 cSt and 25% perfluoroalkyloxyoctane, including early optic disc swelling, retinal edema, and intraretinal inflammation with diffuse narrowing of arteries and veins. The challenges of tamponade research remain the provision of a wider arc of tamponade and long-term intraocular permanence with inertness of the compound while at the same time providing uniform transport of nutrients to intraocular tissue. Despite years of effort, we still remain far from providing good solutions for these “solutions.” S. Donati et al. reported that a promising alternative to the present compounds could be the smart hydrophilic polymers. They are capable of swelling by absorbing its own weight in water, with further possibilities of a thermosetting and with interactive properties with the environment (glucose, glutathione), pH, and light. These properties allow the molecules to be modulated inducing the gelification, better drug diffusion, and increased gel expansion. Perhaps the real solution for an ideal tamponade agent may involve a paradigm shift away from traditional agents.

*Mario R. Romano* *Xun Xu* *Kenneth K. W. Li*

